# Inhalation of phlai-containing essential oil enhances cognition: a comparison with olive oil in healthy adults

**DOI:** 10.3389/fphar.2025.1672991

**Published:** 2026-01-14

**Authors:** Sumittra Gomonchareonsiri, Warangkana Arpornchayanon, Sunee Chansakaow, Pairada Varnado, Nahathai Wongpakaran, Tinakon Wongpakaran

**Affiliations:** 1 Department of Physiology, Faculty of Medicine, Chiang Mai University, Chiang Mai, Thailand; 2 Department of Pharmacology, Faculty of Medicine, Chiang Mai University, Chiang Mai, Thailand; 3 Department of Pharmaceutical Sciences and Medicinal Plant Innovation Center, Faculty of Pharmacy, Chiang Mai University, Chiang Mai, Thailand; 4 Department of Psychiatry, Faculty of Medicine, Chiang Mai University, Chiang Mai, Thailand

**Keywords:** aromatherapy, cognition, functional foods, phlai, sustainable food systems, Zingiber montanum, Zingiber tenuiscapus

## Abstract

**Background/Objectives:**

Phlai (*Zingiber montanum* and *Zingiber tenuiscapus*), traditionally valued for its anti-inflammatory properties, has potential applications as a functional ingredient in sustainable food systems. However, its effects on cognitive function are not well understood. This study investigates the acute cognitive effects of inhaling Phlai-containing essential oil in healthy adults, aiming to explore its potential as a basis for developing plant-based cognitive enhancers within the context of nutritional sustainability.

**Methods:**

A double-blind, randomized, controlled trial was conducted with 40 healthy male volunteers. Participants were assigned to either an olive oil group (n = 20) or an essential oil blend (ESOB) group (n = 20), with Phlai as the primary component. Each participant inhaled a single dose of their respective essential oil for 15 min. Cognitive performance was assessed before and after the intervention using the Montreal Cognitive Assessment (MoCA), the Digit Span Test, the Verbal Fluency Test (VFT), and the Word List Learning Test (WLL). Differences in cognitive outcomes between groups were analyzed using one-way ANCOVA, with a focus on working memory, short-term memory, retrieval fluency, executive function, and global cognition.

**Results:**

Both olive oil and ESOB inhalation resulted in measurable acute improvements in several cognitive domains. Notably, the ESOB Phlai produced significantly greater enhancements in working memory and executive function compared to olive oil. These findings highlight ESOB Phlai’s promise as a functional botanical ingredient with cognitive benefits, suggesting its future value in the development of sustainable, plant-based functional foods or nutraceuticals.

**Conclusion:**

Single-dose inhalation of a Phlai-containing essential oil may acutely enhance cognitive function in healthy adults, supporting the consideration of Phlai as a sustainable, plant-derived ingredient for functional food systems. Further research is warranted to confirm these findings and to investigate practical applications within the food and nutrition sector.

**Clinical Trial Registration:**

https://www.thaiclinicaltrials.org/show/TCTR20180710008, identifier TCTR20180710008.

## 1 Introduction

*Zingiber montanum* (Koenig.) Link ex Dietr., commonly known as Cassumunar ginger or Phlai, and *Zingiber tenuiscapus* are indigenous species widely distributed in Thailand. These rhizomes are rich sources of essential oils, terpenoids, curcuminoids, and phenylbutanoids, compounds known for their analgesic (Chongmelaxme et al., 2017), antimicrobial, antioxidant, and anti-inflammatory properties. While *Zingiber officinale* (common ginger) extract has demonstrated positive effects on working memory in humans (Saenghong et al., 2012) Evidence regarding the cognitive benefits of other Zingiber species, such as Z. montanum, Z. cassumunar, and Z. tenuiscapus, remains scarce.

Aromatherapy, particularly through the inhalation of essential oils, is an emerging approach in functional food systems that utilizes natural, plant-derived compounds to promote health and wellbeing. Essential oils from plants such as bergamot, lavender, cedarwood, yuzu, and rose have been traditionally used in inhalation aromatherapy for their potential neurocognitive effects (Sánchez-Vidaña et al., 2017). Although oral consumption of plant-derived compounds is typical (Arpornchayanon, 2017), inhalation therapy offers a non-invasive, safe, and cost-effective alternative (Arpornchayanon, 2017). Upon inhalation, volatile constituents of essential oils rapidly reach the olfactory epithelium, where they activate chemoreceptors and are indirectly absorbed into circulation through the capillaries of the respiratory mucosa (Tisserand and Balacs, 1995; Clarke, 2008). Olfactory stimulation subsequently triggers neuronal signals to the olfactory bulb and cortex, with downstream effects including the modulation of neurotransmitters such as endorphins, serotonin, and acetylcholine (Kennedy et al., 2018). These signals ultimately influence the limbic system, which is involved in emotional, behavioral, and memory processes (Price and Price, 2012).

Despite promising traditional use and recognized health-promoting properties, there is limited research on the effects of Zingiber spp. Essential oils on cognitive function, particularly in the context of ethnopharmacological traditions and their potential as functional ingredients in cognitive health management. Addressing this knowledge gap may inform the development of plant-based therapeutic strategies designed to enhance cognitive performance.

Previous research has demonstrated cognitive benefits from inhaling various essential oils, including rosemary, lavender, sage, and ylang-ylang, with enhancements in memory, attention, and alertness (Moss et al., 2003; Moss et al., 2008). However, to date, very few studies have examined the effects of essential oils derived from *Z. montanum*, *Zingiber cassumunar*, or *Z. tenuiscapus* on cognitive outcomes; evidence is primarily restricted to preclinical or analgesic/anti-inflammatory applications, with little known about their impact on human cognition. Olive oil was selected as a control due to its neutral sensory profile, as supported by its use as a placebo in prior aromatherapy studies. While olive oil contains minor volatile compounds, it lacks the potent aromatic properties of essential oils and is unlikely to have an independent effect on cognitive function via inhalation (Fazlollahi et al., 2023).

In this study, we investigated the acute effects of inhaling an essential oil blend containing Phlai (ESOB Phlai) (*Z. montanum* and *Z. tenuiscapus*) on cognitive function in healthy adults. Only male participants were included to reduce heterogeneity associated with cognitive and olfactory variances due to hormonal fluctuations during the menstrual cycle (Sorokowski et al., 2019). Based on prior evidence with *Z. officinale* (Saenghong et al., 2012), we hypothesized that acute ESOB Phlai inhalation would enhance working memory and may positively influence a range of cognitive functions—including attention, concentration, language, visuoconstruction, conceptual thinking, calculation, orientation, and executive function—in healthy adults. Olive oil was selected as a comparator due to its mild scent, providing appropriate control for the aromatic properties of the ESOB. This research may contribute to the growing body of knowledge on sustainable, plant-derived ingredients with potential applications in functional foods and nutraceuticals targeting cognitive health.

## 2 Materials and methods

### 2.1 Study design

This study was a double-blind, randomized, parallel-controlled trial conducted at the Clinical Trial Unit, Department of Pharmacology, Chiang Mai University. Approval was obtained from the Research Ethics Committee of the Faculty of Medicine, Chiang Mai University, Thailand.

### 2.2 Essential oil characterization

The ESOB Phlai (Zingiber montanum and Zingiber tenuiscapus) was analyzed using gas chromatography-mass spectrometry (GC-MS). Chemical constituents were identified using the NIST Library and Kováts retention indices, with standard alkanes (C8–C20) as references. Major compounds included alpha-terpinolene (19.27%), p-cymene (12.04%), alpha-terpinene (11.11%), sabinene (10.95%), terpinene-4-ol (9.31%), and 1,8-cineole (7.38%). The physical and chemical stability of ESOB was confirmed before use.

### 2.3 Participants

Forty healthy male volunteers, aged 18–25 years (mean education: 14.87 years), were recruited.

All participants underwent a physical examination, including vital signs, and a basic neurologic examination to confirm the absence of acute or chronic illness. All participants underwent a physical and neurological examination by the researcher (A.W.), a board-certified otolaryngologist. Normal nasal anatomy was defined as the absence of structural abnormalities, including septal deviation, nasal polyps, or other lesions, as confirmed by anterior rhinoscopy and flexible nasoendoscopy. Adequate olfactory function was established using a standardized smell test, in which participants correctly identified a set of common odorants, confirming intact olfactory capability.

Exclusion criteria included active sinonasal disorders, use of CNS depressants or stimulants (within 2 weeks), and psychiatric or neurological conditions. Written informed consent was obtained. No participants were excluded after the physical examination. All enrolled volunteers met the eligibility criteria following screening.

### 2.4 Sample size and randomization

We use parameters from the most related research of Zingiber on cognitive function for sample size calculation (Saenghong et al., 2012). The sample size was calculated for testing two dependent means (two-tailed test). Determining the significance level (α) at 0.05, statistical power (1-ß) of 0.80 (Chow et al., 2003). The difference in means of the primary outcome (working memory) from previous research was 8.25. The standard deviations for group 1 and group 2 were 6.68 and 8.26, respectively. The ratio of sample sizes in Group 1 to Group 2 was 1:1. This yielded a total sample of 30 participants, with 15 in each group. We enrolled 20 participants per group to increase power and to compensate for potential drop-out (Lancaster et al., 2004).

**FIGURE 1:**
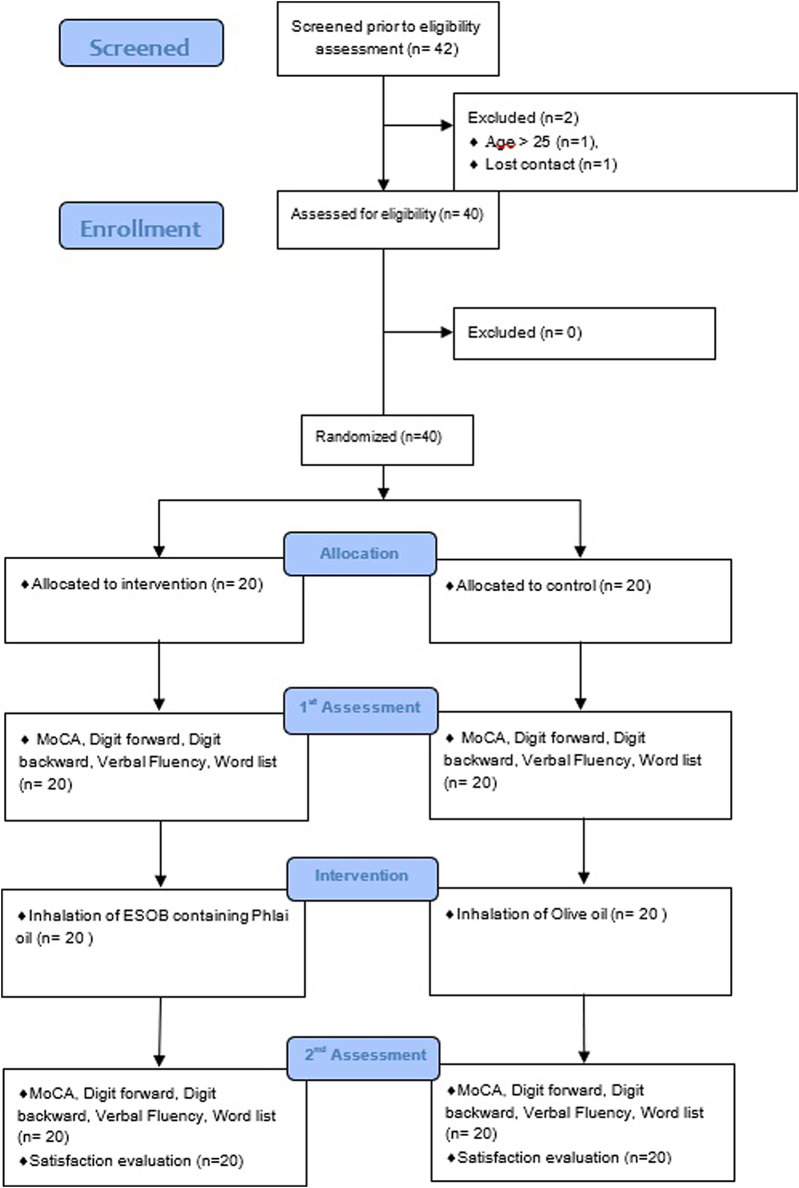
A CONSORT flow diagram this figure depicts participant recruitment and study completion.

To account for attrition, 20 participants were enrolled per group. Participants were randomized in equal numbers to the ESOB or control group (olive oil) using computer-generated block randomization (Figure 1).

### 2.5 Procedure

Participants attended a screening session during which eligibility was confirmed and informed consent obtained. On the testing day, participants arrived and were randomized, then completed baseline cognitive assessments (15 min before intervention). They then inhaled either ESOB or control oil for 15 min in a dedicated room, followed by immediate post-intervention cognitive assessments (within 5 min after intervention). Assessment and procedures in total required approximately 50–60 min per participant.

### 2.6 Interventions

Participants inhaled either ESOB or 100% olive oil (control) diluted in hot water (0.75 mL oil: 500 mL, 85 °C) via steam vaporization for 15 min. Steam inhalation was chosen to facilitate the efficient delivery of volatile aromatic compounds from oils, as steam acts as a carrier to enhance olfactory exposure. This approach is commonly employed in aromatherapy research to ensure consistent administration of volatile constituents. The inhalation procedure was performed at a distance of 12 cm from the vessel, with participants’ eyes closed and protected by eye pads to avoid ocular irritation. Sessions were conducted in isolated, well-sealed rooms to prevent aroma cross-contamination; research assistants ensured that participants did not interact with one another before or after the intervention. Double blinding was maintained using separate intervention rooms and procedures, and cognitive assessments were conducted by raters blinded to group allocation. Sessions were conducted in isolated, well-sealed rooms to prevent aroma cross-contamination; research assistants ensured that participants did not interact with one another before or after the intervention. Double blinding was maintained through separate intervention rooms and procedures; cognitive assessments were conducted by raters blinded to group allocation.

The carrier for the intervention was distilled water prepared on-site using the laboratory’s standard distillation process. The delivery system for steam inhalation consisted of standard laboratory beakers and glassware (not a commercially branded inhalation device). No specific supplier was applicable for either the carrier or the delivery system.

### 2.7 Outcomes and assessments

The primary outcome was change in working memory, assessed by the Verbal Fluency Test (VFT), Word List Learning Test (WLL), and Montreal Cognitive Assessment (MoCA). Secondary outcomes included attention and concentration (Digit Span), language, visuoconstruction, conceptual thinking, calculation, orientation (MoCA), and executive function (VFT, MoCA).

- Montreal Cognitive Assessment (MoCA): Assesses 11 cognitive domains, including concentration, attention, executive function, memory, language, visuoconstruction, conceptual thinking, orientation, and calculation; a score ≥25 indicates normal cognition (Nasreddine et al., 2005; Hemrungrojn, 2011; Tangwongchai, 2009).
- Digit Span Test (Wechsler Memory Scale): Digit Span (forward and backward) was used to assess attention and working memory (The Psychological Corporation, 1997).
- Verbal Fluency Test (VFT): Measures semantic and phonemic fluency, attention, working memory, and executive function. In this study, participants were asked to name as many words as possible within a specific category—animals (Semantic Fluency)—within a set time. Then, the participant was asked to generate as many words as possible beginning with a specific letter (e.g., “F,” “S,” or “P”) within the same time limit (Phonemic Fluency) (Reitan, 1985; Bertola et al., 2014; Shao et al., 2014; Henry and Crawford, 2004; Takács et al., 2014).
- Word List Learning Test (WLL): Assesses immediate and delayed recall. The participant was asked to remember a list of 10 words that they had read, followed by testing their free recall of the list after a delay (Morris et al., 1989).

Cognitive tests were administered 15 min before and after the intervention. A visual analog scale was used to assess participant satisfaction, and adverse events were recorded.

### 2.8 Statistical analysis

Descriptive statistics (mean, standard deviation, and percentage) summarize demographic and outcome variables. Paired t-tests compared within-group pre- and post-intervention differences. ANCOVA (adjusting for baseline scores) compared between-group effects. Normality was evaluated using Kolmogorov-Smirnov and Shapiro-Wilk tests. For non-normal MoCA distributions, Quade’s rank ANCOVA with generalized estimating equations was used. A p-value <0.05 indicated statistical significance (Van Breukelen, 2006; Fan and Zhang, 2017).

## 3 Results

A total of 40 participants were randomized into two groups: ESOB Phlai (n = 20) and olive oil (n = 20). The groups were comparable in age, weight, height, years of education, and baseline vital signs. Table 1 presents the baseline characteristics of the participants. No significant differences were observed, confirming that the groups were well matched at baseline.

**TABLE 1:** Participant characteristics.

Variable	All (n = 40)	ESOB Phlai (n = 20)	Olive (n = 20)	t-statistics	p-value
Age (years)	22.11 (2.12)	21.80 (2.20)	22.20 (2.10)	0.59	0.56
Weight (kg)	68.20 (13.13)	66.65 (11.30)	69.75 (14.80)	0.74	0.46
Height (cm)	172.53 (5.88)	173.00 (6.10)	172.05 (5.80)	0.51	0.62
Years of education	14.82 (1.72)	14.55 (1.40)	15.10 (1.90)	1.01	0.32
Systolic BP (mmHg)	117.25 (12.55)	116.85 (13.12)	117.65 (12.24)	0.19	0.85
Diastolic BP (mmHg)	74.70 (7.82)	74.10 (8.07)	75.30 (7.67)	0.5	0.62
Pulse (bpm)	75.13 (8.11)	74.80 (8.26)	75.45 (8.14)	0.25	0.8
Respiratory rate	17.05 (1.26)	17.10 (1.28)	17.00 (1.28)	0.26	0.79

Data are presented as mean (SD). No significant differences were found between groups (all p > .05).

Table 2 shows within-group pre- and post-test comparisons for cognitive function. Both ESOB Phlai and Olive Oil groups showed significant improvement in MoCA scores (mean difference: 1.20 ± 1.54, p = 0.003, and 1.15 ± 1.60, p = 0.005, respectively) and in Word List Learning (WILL; 0.65 ± 0.99, p = 0.008 for ESOB Phlai; 0.95 ± 1.44, p = 0.009 for Olive Oil). Only the ESOB Phlai group had significant gains in Digit Backward (1.05 ± 1.85, p = 0.020) and Scale Digit (0.95 ± 1.85, p = 0.028). For VFT (phonemic A), improvement was significant in both groups but greater for ESOB Phlai (6.90 ± 4.42, p < 0.001) compared to Olive Oil (2.40 ± 3.87, p = 0.012). Changes in VFT animal, VFT (S), and Digit Forward were not significant, except for VFT (P), where only Olive Oil showed improvement (2.10 ± 3.26, p = 0.010). Overall, both ESOB Phlai and Olive Oil groups exhibited pre-to-post gains across several cognitive measures, with the ESOB Phlai group showing particularly marked improvement in verbal fluency on the phonemic A task.

**TABLE 2:** Comparison of pre- and post-test of cognitive function tasks between ESOB phlai and olive.

Test	Group	Pre-test		Post-test		Mean difference (SD)	95%CI		*t*	p-value
		Mean	SD	Mean	SD		Lower	Upper		
MoCA	ESOB phlai	27.45	1.93	28.65	1.39	−1.20 (1.54)	−1.92	−0.48	−3.48	0.003
	Olive	27.20	1.67	28.35	1.23	−1.15 (1.60)	−1.90	−0.40	−3.22	0.005
DF	ESOB phlai	12.20	2.29	12.50	2.44	−0.30 (1.32)	−0.93	0.33	−1.00	0.330
	Olive	12.60	1.98	13.20	2.04	−0.60 (1.64)	−1.37	0.17	−1.64	0.117
DB	ESOB phlai	8.10	2.97	9.15	2.98	−1.05 (1.85)	−1.92	−0.19	−2.54	0.020
	Olive	8.00	2.96	8.20	2.53	−0.20 (2.33)	−1.29	0.89	−0.38	0.705
Scale digit	ESOB phlai	12.10	3.35	13.05	3.56	−0.95 (1.85)	−1.79	−0.11	−2.37	0.028
	Olive	12.25	2.90	13.25	3.04	−1.00 (2.64)	−2.23	0.23	−1.70	0.106
VFT animal	ESOB phlai	23.45	6.61	23.55	4.54	−0.10 (4.10)	−2.02	1.82	−0.11	0.914
	Olive	24.70	4.74	22.60	5.55	2.10 (5.21)	−0.34	4.54	1.80	0.087
VFT (A)	ESOB phlai	14.25	5.84	21.15	5.81	−6.90 (4.42)	−8.97	−4.83	−6.98	0.000
	Olive	15.65	4.66	18.05	3.87	−2.40 (3.87)	−4.21	−0.59	−2.77	0.012
VFT (S)	ESOB phlai	14.10	6.29	14.20	5.68	−0.10 (4.81)	−2.35	2.15	−0.09	0.927
	Olive	15.40	4.20	14.85	4.61	0.55 (3.82)	−1.24	2.34	0.64	0.527
VFT (P)	ESOB phlai	14.05	5.77	14.90	4.90	−0.85 (3.82)	−2.64	0.94	−1.00	0.332
	Olive	13.30	5.06	15.40	5.33	−2.10 (3.26)	−3.63	−0.58	−2.88	0.010
WILL	ESOB phlai	7.90	1.22	8.55	1.07	−0.65 (0.99)	−1.11	−0.19	−2.94	0.008
	Olive	7.78	1.53	8.73	0.99	−0.95 (1.44)	−1.63	−0.27	−2.93	0.009

ESOB, essential oil blend; MoCA, montreal cognitive assessment; DF, digit forward; DB, digit backward; VFT, verbal fluency test; WILL, word list learning test.

An ANCOVA was conducted to examine the effect of the intervention (ESOB Phlai vs. Olive Oil) on post-intervention outcomes. No significant treatment effects were observed for any measures except the VFT (Phonemic A) scores. Therefore, only the results for VFT (Phonemic A) are presented.

Table 3 presents the treatment effect on VFT (Phonemic A) scores, controlling baseline performance (pre-test VFT-A), age, weight, height, and years of education. All statistical assumptions for ANCOVA were met. The overall model was significant, F (6, 33) = 6.12, p < 0.001, accounting for approximately 53% of the variance in post-intervention VFT (Phonemic A) scores (R^2^ = 0.53; adjusted R^2^ = 0.44). Age (B = −0.11, p = 0.748), weight (B = 0.03, p = 0.609), height (B = −0.15, p = 0.286), and years of education (B = −0.13, p = 0.768) were not significant predictors, as all confidence intervals included zero. In contrast, baseline VFT scores (PrePhonemicA) were strongly associated with post-intervention performance (B = 0.67, SE = 0.14, t = 4.75, p < 0.001, 95% CI [0.38, 0.96], partial η^2^ = 0.41), indicating that higher baseline scores predicted higher post-test outcomes.

**TABLE 3:** Tests of between-subjects effects for post-intervention VFT (phonemic A).

Parameter	B	Std. Error	t	Sig	95% confidence interval		Partial eta squared
					Lower bound	Upper bound	
Intercept	40.172	22.066	1.820	0.078	−4.723	85.066	0.091
Age	−0.110	0.340	−0.324	0.748	−0.802	0.582	0.003
Weight	0.031	0.060	0.516	0.609	−0.091	0.153	0.008
Height	−0.152	0.140	−1.085	0.286	−0.438	0.133	0.034
Years of education	−0.128	0.430	−0.297	0.768	−1.002	0.746	0.003
Pre-test phonemic A	0.670	0.141	4.752	0.000	0.383	0.956	0.406
Treatment (group)	−4.164	1.316	−3.163	0.003	−6.842	−1.486	0.233

**FIGURE 2:**
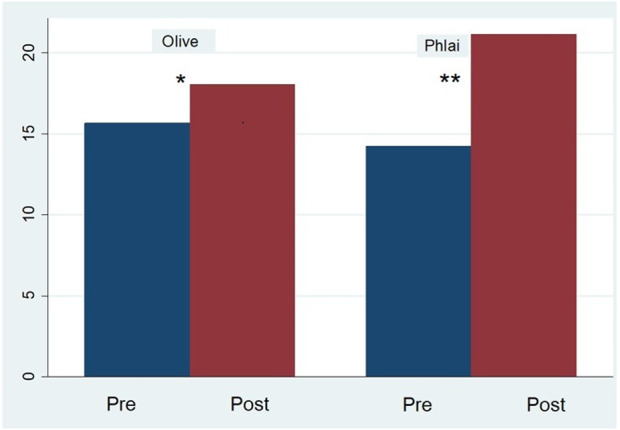
Mean Verbal Fluency Test Scores (A) before and after the intervention. Within-group effects: olive, *p* = 0.012; ESOB phlai, *p* < 0.001. Between-group effect (post-intervention): *p* = 0.002.

Regarding treatment effects, participants in the control group (Olive Oil) scored significantly lower than those in the reference group (ESOB Phlai) on post-intervention VFT (Phonemic A) (B = −4.16, SE = 1.32, t = −3.16, p = 0.003, 95% CI [−6.84, −1.49], partial η^2^ = 0.23), suggesting superior performance in the experimental group after adjusting for covariates. The results are also illustrated in the bar graph (Figure 2).

The satisfaction level of ESOB Phlai oil was significantly higher than that of olive oil, although the difference was not statistically significant (VAS score: 83.40 ± 11.10 vs. 81.45 ± 11.70). No serious adverse events (AEs) were found in either group. All AEs were mild (e.g., dizziness, drowsiness, nausea, nasal discomfort), occurred during or immediately (<5 min) following the 15-min inhalation procedure, and resolved spontaneously within an hour. No protocol adjustments were necessary as all symptoms abated without intervention. All AEs were documented and reported to the study’s principal investigator; medical personnel were present on-site throughout, although no medical intervention was required. The dose of ESOB Phlai or olive oil was not adjusted throughout the study, consistent with safety parameters in prior aromatherapy research. Similar event rates in both groups suggest the method of steam inhalation, rather than the essential oil, contributed to these mild effects.

## 4 Discussion

This randomized controlled trial is among the first to investigate the acute cognitive effects of inhaling a Phlai-containing essential oil blend (ESOB Phlai) in healthy adults. Following a single exposure, both the ESOB Phlai and olive oil groups exhibited measurable improvements in global cognitive function and verbal memory. Notably, participants receiving ESOB Phlai demonstrated greater or distinct enhancements in domains related to working memory, complex attention, and specific aspects of verbal fluency. The observed cognitive gains in the olive oil group require careful consideration. While olive oil is often regarded as a neutral placebo, its use in aromatherapy studies may not be entirely inert.

Interestingly, cognitive improvements were also observed in the olive oil group. These effects may be partially attributable to non-specific influences, such as participant expectations or the calming nature of the intervention setting. Although olive oil is typically considered neutral, it may offer minimal olfactory stimulation capable of eliciting mild shifts in mood or cognitive arousal. Supporting this possibility, a recent systematic review of 11 studies—including RCTs, cohort, and cross-sectional designs—found that mild olfactory properties of olive oil, combined with participant expectations and the controlled, calming research environment, may have contributed to placebo-like effects. Additionally, recent clinical evidence has linked olive oil consumption to reduced cognitive decline, especially in older adults (Fazlollahi et al., 2023). While this trial did not assess ingestion, the possibility that subtle olfactory or psychological mechanisms play a role should not be overlooked. Placebo responses are common in cognitive intervention research, as participants may become more alert or relaxed due to the anticipation of improvement, the soothing study environment, or simply the process of inhalation (Foroughi et al., 2016). Such effects support the critical importance of rigorous placebo selection and standardized research settings in aromatherapeutic investigations.

In contrast, the more substantial cognitive improvements seen following ESOB Phlai inhalation may reflect bioactive actions specific to its phytochemical constituents. Preclinical research on Zingiber species (including Phlai) has consistently demonstrated cognitive benefits—particularly enhancements in learning, memory, and executive function—through modalities such as acetylcholinesterase inhibition, anti-inflammatory, and antioxidant activity (Nakai et al., 2016; Matsui et al., 2012; Okonogi and Chaiyana, 2012). Our GC-MS analysis identified key compounds such as alpha-terpinolene, p-cymene, and sabinene, which have also been linked to improvements in neurocognitive performance in studies with rosemary and lemon balm essential oils (Kennedy et al., 2018). Mechanistically, these terpenoid compounds may modulate neurotransmitter pathways, such as those of acetylcholine, dopamine, and serotonin, especially in brain regions linked to memory and emotion regulation (e.g., limbic and hypothalamic structures) (Gotlib and Joormann, 2010; LeMoult and Gotlib, 2019; Ashby et al., 1999; Ali et al., 2015). Enhancing cholinergic function and mitigating oxidative stress are recognized strategies for preventing mild cognitive impairment and dementia, lending plausibility to our findings.

Moreover, the study’s design—with short-term, controlled exposures in a restful environment—may have synergistically enhanced the observable cognitive benefits. Calming environments can optimize attentional resources and suppress competing sensory distractions, supporting transient boosts in cognitive performance (Taylor et al., 2015). This context mirrors real-world applications, where essential oil inhalation is often practiced in relaxing settings for maximal effect.

Despite these promising results, several limitations warrant consideration. Blinding was not formally assessed; although efforts were made to standardize conditions (e.g., separate rooms, eye pads, blind raters), we did not implement a ‘treatment guess’ procedure or ask participants to identify their group. This remains a limitation, as the distinct aroma of essential oils may have influenced participant expectations. A further limitation of this study is that no training or familiarization session on the cognitive tests was given prior to the pre-intervention assessment. Practice effects—whereby repeated exposure to cognitive tasks leads to improved scores—may have contributed to the post-intervention improvement observed in both groups. Although standard instructions were provided, a pre-test familiarization could have helped control for this effect. Future studies should consider including a training session to minimize practice-related gains and further clarify the specific effects of the interventions. Additionally, the sample was limited to healthy young male adults, restricting the generalizability of our findings. Future studies should include female participants, older adults, and individuals with cognitive impairments. Mood effects, which may influence cognitive performance, were not directly measured; subsequent research should incorporate assessments of mood and subjective alertness.

In summary, our findings provide early evidence that inhalation of a Phlai-based essential oil blend may yield acute improvements in cognitive function beyond those obtained with olive oil alone, likely through both neurobiological and psychological mechanisms. Continued investigation into the phytochemistry, dosing, duration, and real-world applicability of Phlai and related essential oils is warranted, especially for populations at risk of cognitive decline.

## Data Availability

Raw data can be accessed at https://doi.org/10.6084/m9.figshare.3098549. Further inquiries can be directed to the corresponding author.
