# Supplementary figures and images for: Inhalation of phlai-containing essential oil enhances cognition: a comparison with olive oil in healthy adults
Source: Front Pharmacol. 2026 Jan 14;16:1672991. doi: 10.3389/fphar.2025.1672991 (PMC12847393; doi:10.3389/fphar.2025.1672991)

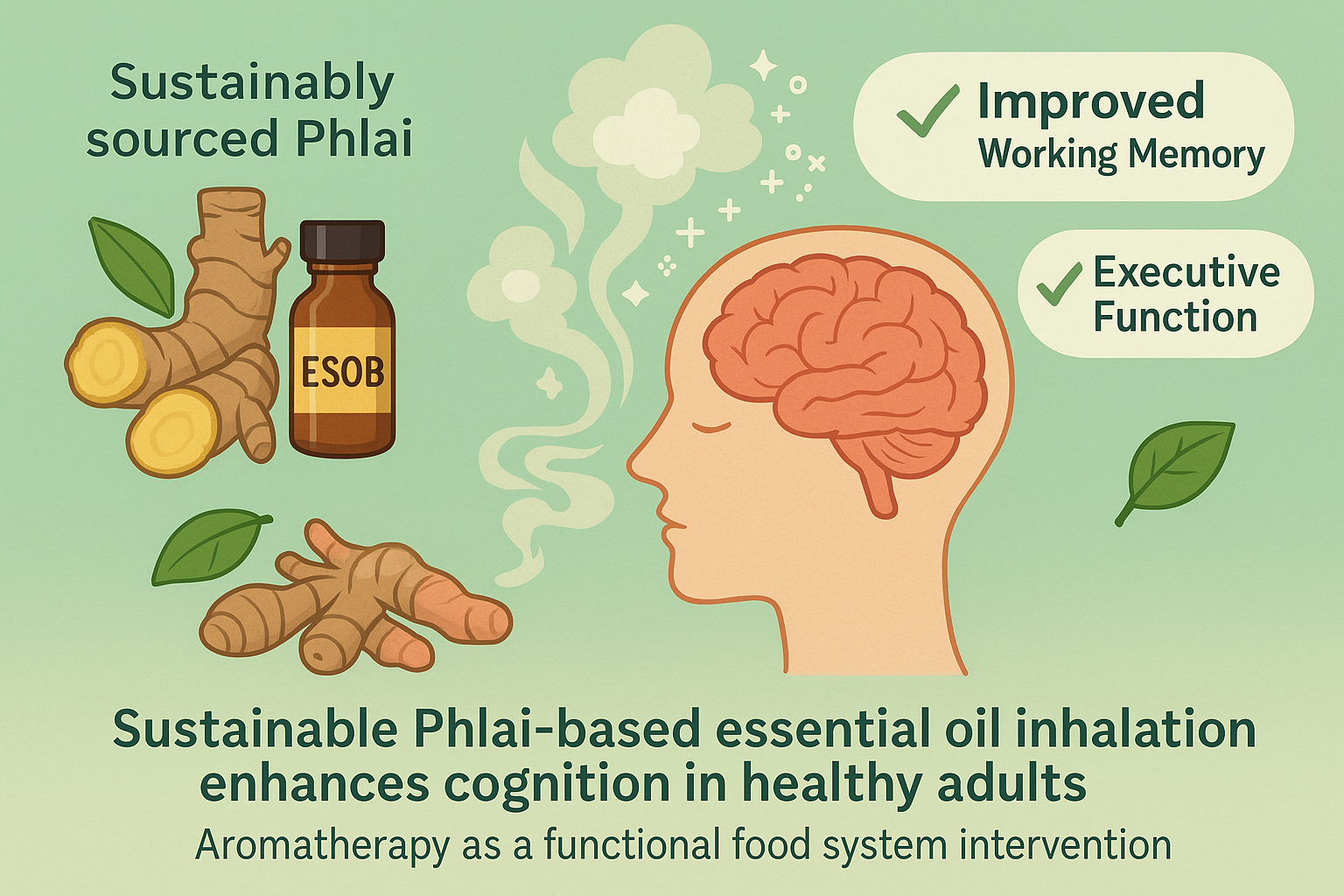

Supplement: Supplementary file 1 [file Image1.png]
